# EEG-Based Cognitive Control Behaviour Assessment: an Ecological study with Professional Air Traffic Controllers

**DOI:** 10.1038/s41598-017-00633-7

**Published:** 2017-04-03

**Authors:** Gianluca Borghini, Pietro Aricò, Gianluca Di Flumeri, Giulia Cartocci, Alfredo Colosimo, Stefano Bonelli, Alessia Golfetti, Jean Paul Imbert, Géraud Granger, Railane Benhacene, Simone Pozzi, Fabio Babiloni

**Affiliations:** 1 Dept. of Molecular Medicine, Sapienza University of Rome, Piazzale Aldo Moro, 5, 00185 Rome, Italy; 2 BrainSigns srl, via Sesto Celere, 00152 Rome, Italy; 3 IRCCS Fondazione Santa Lucia, Neuroelectrical Imaging and BCI Lab, Via Ardeatina, 306, 00179 Rome, Italy; 4 Dept. of Anatomical, Histological, Forensic & Orthopedic Sciences, Sapienza University of Rome, Piazzale Aldo Moro, 5, 00185 Rome, Italy; 5 DeepBlue srl, Piazza Buenos Aires 20, 00185 Rome, Italy; 6 École Nationale de l’Aviation Civile, 7 Avenue Edouard Belin, 31000 Toulouse, France

## Abstract

Several models defining different types of cognitive human behaviour are available. For this work, we have selected the *Skill, Rule and Knowledge* (SRK) model proposed by Rasmussen in 1983. This model is currently broadly used in safety critical domains, such as the aviation. Nowadays, there are no tools able to assess at which level of cognitive control the operator is dealing with the considered task, that is if he/she is performing the task as an automated routine (*skill* level), as procedures-based activity (*rule* level), or as a problem-solving process (*knowledge* level). Several studies tried to model the SRK behaviours from a *Human Factor* perspective. Despite such studies, there are no evidences in which such behaviours have been evaluated from a neurophysiological point of view, for example, by considering brain activity variations across the different SRK levels. Therefore, the proposed study aimed to investigate the use of neurophysiological signals to assess the cognitive control behaviours accordingly to the SRK taxonomy. The results of the study, performed on 37 professional *Air Traffic Controllers*, demonstrated that specific brain features could characterize and discriminate the different SRK levels, therefore enabling an objective assessment of the degree of cognitive control behaviours in realistic settings.

## Introduction

The efficiency of humans in coping with complex situations is largely due to the availability of a large repertoire of different mental representations of the environment from which rules to control behaviour can be generated *ad-hoc* ^1–3^. Nowadays, several models defining the different types of cognitive human behaviour are available^4–6^. For this study, we have selected the *Skill, Rule and Knowledge* (SRK) model proposed by Rasmussen in 1983^7^. Accordingly with basic different ways of representing the constraints in the cognitive human behaviour, three typical levels emerge: *skill-*, *rule-*, and *knowledge-based* level^7, 8^.

The *skill-based* behaviour represents sensory-motor performance during acts or activities which, following a statement of an intention, take place without conscious control as smooth, automated, and highly integrated patterns of behaviour. In most skilled sensory-motor tasks, the body acts as a multivariable continuous control system, synchronizing movements depending on the surrounding environment. Performance is based on feedforward control and depends on a very flexible and efficient dynamic internal world model. Characteristically, skilled performance rolls along without conscious attention or control. The total performance is smooth and integrated, and sense input is not selected or observed: the senses are only directed towards the aspects of the environment subconsciously needed to update and orient the internal map. In general, human activities can be considered as a sequence of such skilled acts or activities composed for the actual occasion. The flexibility of skilled performance is due to the ability to compose, from a large repertoire of automated subroutines, the sets suited for specific purposes.

At the level of *rule-based* behaviour, the composition of such a sequence of subroutines in a familiar working situation is typically controlled by a stored rule or procedure, which may have been empirically derived during previous occasions, or it may be prepared in correspondence of conscious problem-solving and planning. In this case, the performance is goal-oriented but structured by “feedforward control” through stored rules. Very often, the goal is not even explicitly formulated, but it is implicitly found from previous successful experiences. Feedback correction during performance will require functional understanding and analysis of the current response of the environment, which may be considered as an independent concurrent activity at the higher level (*knowledge-based*).

The boundary between skill-based and rule-based performance is not very distinct, and much depends on the level of training and the attention of the person. In general, the skill-based performance rolls along without the person’s conscious attention, and he/she will be unable to describe how he/she controls and on what information he/she bases the performance. The higher level, rule-based coordination, is generally based on explicit know-how, and the adopted rules can be reported by the person. During unfamiliar situations, facing with an environment for which no *know-how* or rules for control are available from previous experiences, the control of the performance must move to a higher conceptual level, in which the performance is goal-controlled and *knowledge-based*. In this situation, the goal is explicitly formulated, based on an analysis of the environment and the overall aims of the person. If we consider that in real life people have a varying degree of expertise when performing a task depending on variations and disturbances, the correspondence with the three cognitive control behaviours in the present context is clear. One aspect of the categorization of human performance in *skill/rule/knowledge*-based behaviour is the role of the information derived from the environment, which is basically different in the various categories. This is the case of unfamiliar situations, when same information or events could be perceived in many different ways depending on the operator’s expertise level^8^. As described by Reason^9^, each level has its own pros and cons, and it is related to specific types of performance and error: for instance, as the skill level is characterised by a low level of control, the corresponding behaviour is fast and effortless, but it is also related to slips and lapses errors. While performing at the rule-based level, it is possible to quickly and effectively face familiar and anticipate unfamiliar events, but rule-based mistakes can happen, such as the misapplication of right rules or the application of wrong rules. Finally, the *knowledge-based* behaviour leads to the resolution of new problems, to the generation of new rules and, in general, to innovation and creativity; on the other hand, this behaviour is slow, cognitively demanding and very prone to error. The reason of the SRK model choice was that the proposed study has been carried out for safety critical systems, with particular interest in the *Air Traffic Management* (ATM) domain, where the *Human Factor* (HF) bearing has been deeply investigated also from a neurophysiological point of view^10–20^. The SRK framework is broadly known and applied in many operational environments, as aviation and healthcare, for the design^21^ and generation of the human error taxonomy^22^. A second reason for our choice was related to the origin of this model, as it has not been developed from cognitive psychology studies, but from the direct observation of operators performing their working tasks in complex systems. The use of a neurophysiological-based model, rather than a psychological one, poses some challenges. In fact, in the scientific literature there are no direct links with the well-known cognitive psychology concepts, and this study can contribute to better highlight the potentiality of the SRK model and the proposed methodology. As mentioned above, the SRK framework is still lacking of a quantitative, reliable and validated way to be quantified. Assessing the level of the cognitive control behaviour of professional personnel in operational environments would enable a deeper comprehension of the cognitive processes actually activated in real work activities.

## Toward an integration between srk and neurophysiological variables

Several interesting issues about the neuroanatomical and functional models may be related to the level of attentional control of SRK framework^7^ for a possible integration between SRK and neurophysiological variables. One of the first models of attentional control has been proposed by Posner and Petersen^23^ and it consists of two separate, but closely interrelated, systems: an anterior attentional system (linked to environmental monitoring and detection of target stimuli), and a posterior attentional system (linked to the orientation of attention). More recent versions of this model describe three systems^24–26^. In particular, the *Alert/vigilance system* (connected to frontal and parietal regions), the *Orienting system* (consisting in posterior parietal, frontal cortex, *temporo-parietal junction* (TPJ), thalamic nuclei, and superior colliculus), and the *Executive system* (consisting in prefrontal medial cortex, anterior cingulate cortex, and supplementary motor area). Furthermore, recent studies^27^ have proposed that cortical control of attention is splitted into two functionally-divided but interacting systems:

- *Dorsal frontoparietal network* (top-down, endogenous attention) is guided by cognition, involved in control of goal-driven attention, and preparation and application of relevant stimuli selection.
- *Ventral frontoparietal network* (bottom-up, exogenous attention) is guided by perception and it is a stimulus-driven attentional system. It is involved in disengagement and re-orienting of attention towards salient or unexpected stimuli.

The SRK framework can be connected to the attentional model mentioned above towards the investigation of neurophysiological correlates of Rasmussen’s taxonomy. In fact, among the cognitive neuropsychologists, in terms of limited capacity, it has been hypothesized a mechanism with the function of programming and controlling cognitive processes in relation to priorities, goals and external conditions. This mechanism, called *Central Executive*, provides a higher-level processing in a hierarchical organization. Since the central executive has a limited capacity, many routine operations should be delegated to mechanisms that operate automatically, regardless of the voluntary attentional costs in terms of effort. An automatic process^28^ consists of the activation of a learned sequence of elements without the conscious control. A controlled process instead requires continuous effort and monitoring by the subject. It works in serial mode, drawing on the stock of the short-term memory. The automation is facilitated by practice, which makes the process faster, parallel, with less mental load demand. However, it should be remembered that attention does not always coincide with a controlled process: the automatic process also focuses attention on certain stimuli, selecting among other not-relevant stimuli for the task, and keeps focusing on them for the necessary time. Neumann^29^ pointed out that the automatic process is not completely uncontrolled, but rather control is below the threshold of consciousness. Therefore, it seems appropriate to distinguish between *non-intentional* attentional processes, involving minimal effort and self-perception of the subject, and *intentional attention*, conscious and characterized by a limited capacity. Several empirical studies^30–33^ demonstrated how information not consciously receiving attention can be processed in relation to their meaning, and in absence of intentional control. Since attentional mechanisms can control the access to awareness^34, 35^, different models tend to identify the function of attentional control with the conscience, such as central operative system of activation, and self-monitoring. According to Allport^30^ and Schmidt^36^, intentionality, attention, control and awareness are the constitutive dimensions of consciousness. To sum up, the attentional level is very important for the neurophysiological characterization and discrimination of the SRK behaviours in the ATM domain^4, 37^, since *skill-based* behaviour generally rolls along without the person’s conscious attention, whereas higher levels, *rule-* and *knowledge-based* behaviours, are generally based on explicit attentional control. In other words, higher activations of the *Electroencephalographic* (EEG) features correlated to the attentional level will represent higher attention demand, therefore relative to *rule-* and *knowledge-based* behaviours.

Hence, the objective of this study was to assess whether it was possible to differentiate the three degrees of cognitive controls proposed by the SRK model^7^ by the analysis of the Air Traffic Controllers’ brain activity during the execution of a high realistic ATM scenario. In particular, we aimed to characterize the three cognitive control behaviours in terms of “pure” cognitive engagement by considering only brain features linked to cognitive processes, such as information processing, decision making, working memory, and attention. In fact, we have not considered motor brain features (e.g. sensory-premotor cortex) in order to do not differentiate the SRK behaviours on the basis of different amount of movements (hands or feet).

## Material and Methods

### Cognitive functions and related brain features correlated to the SRK levels

**Figure 1:**
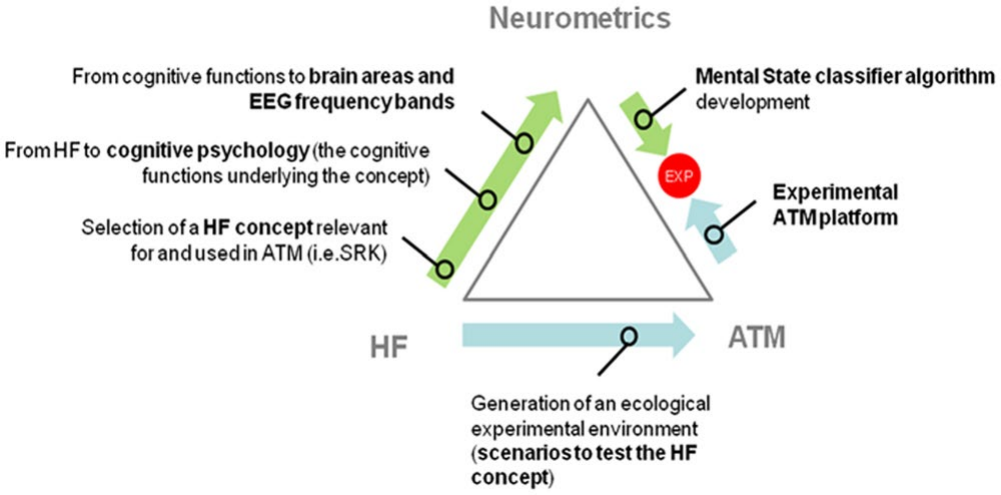
Simplified illustration of the multi-disciplinary approach used in the study. Three levels of analysis have been considered: human factor concepts, realism of the experimental task, and neurophysiological evidences related to the main cognitive phenomena linked to the SRK behaviours.

In order to define a complete overview of the considered topic, a multidisciplinary approach has been used. Such approach is summarised in Fig. 1.

Firstly, a literature review has been specifically done to find out HF concepts relevant for and used in the ATM field. Then, such HF concepts have been translated into psychophysiological functions to finally identify the brain features underlying cognitive processes involved in the SRK behaviours (green arrows). In other words, we sought the main cognitive processes involved in the three levels of cognitive control behaviours, and then we selected the EEG rhythms by which objectively describe such different behaviours. At the same time, an experimental ATM environment has been set up in order to induce the behaviours we wanted to assess in the Controllers (light blue arrows). Finally, experiments have been conducted in order to validate our hypothesis and verify that the selected brain features were capable of discriminating the three levels of cognitive control behaviour.

By definition, the three behaviours involve different cognitive processes and levels of automatism, and they correspond to operational conditions which require different amount of cognitive effort. For example, as quoted previously, the *skill* behaviour needs automatic actions. During *rule* behaviour, the user has to identify the right procedures and then apply them. In other words, the *rule* behaviour can be roughly defined as long-term memory access + automatic actions (*skill behaviour*). During *knowledge* behaviour, the user has to understand what is going on, organize and process the information, plan a strategy, find out the right procedures, and finally to apply them. This behaviour can be seen as information processing + decision making + procedures selection (*rule* behaviour) + automatic actions (*skill* behaviour). Therefore, the cognitive control behaviours, especially the *rule* and *knowledge* ones, are slightly overlapped to each other, and even if they were designed as pure as possible, they would always show some contributes derived from the others. In order to investigate the possibility to measure and quantify SRK events in realistic settings, we properly inserted the SRK events within ATM conditions of different complexity, where the difficulty profile of the task changed over time to simulate realistic air-traffic situations. Therefore, the issue could be to not being able to discriminate SRK behaviours when the difficulty of the faced condition changes (i.e. different workload demand). In this regard, the mental workload is a complex concept and it involves many cognitive processes, such as stress, attention, time pressure, task difficulty, emotional status, mental effort, physical demand, memory, etc^38^. For such a reason, the proposed work aimed to find out brain features able to characterize the three cognitive control behaviours (*Skill*, *Rule*, and *Knowledge*) even if the task difficulty (or workload demand) was changing. Brain activations are generally correlated to specific cognitive phenomena^39^. In fact, literature evidences show that an increase of EEG activity, especially in the theta band over the frontal cortex has been observed when the demand of executive control (attention and working memory)^40–42^, the activation of decision-making processes (like resolution of conflicts and error detection^43^), problem solving demand^44^, mental workload request^38, 45–51^, and the task complexity are high^16, 17, 19, 52^. Additionally, theta rhythms, engaged in the hippocampus/PFC (prefrontal cortex), potentially interplays in the consolidation of memory and empirical results reports the effect of enhanced theta oscillations on memory consolidation^53–57^, and induction of long-term plasticity^58–60^. Therefore, we hypothesized that enhanced parietal theta activation supports exchange of information between the hippocampus and neocortical areas, hence it could be considered as an indicator of memory consolidation^61, 62^.

Concerning the alpha EEG rhythm, the alpha-band frequency range has been usually associated with attention^63–65^. A measure of support for this notion comes from imaging studies reporting attentional modulations in the pulvinar nucleus^66–68^ and in the lateral geniculate nucleus^69, 70^. In the work of Neuper and Pfurtscheller^71^, it has been shown that the *event-related desynchronization* (ERD) of EEG activity in the alpha band reflected an increased excitability level of neurons in the involved cortical areas, which could be related to an enhanced information transfer in thalamo-cortical circuits. In contrast, *event-related synchronization* (ERS) of alpha activity (i.e. increases in alpha activity) is thought to reflect a reduced state of active information processing in the underlying neuronal networks^72^, reflecting the inhibitory aspect of alpha-band oscillations^73, 74^.

Klimesch^75^ argued that the two fundamental functions of attention, *suppression* and *selection*, enable selective access to the *Knowledge System* (KS) and operate accordingly to the proposed inhibition timing function of alpha-band activity^76, 77^. The KS is a storage system comprising not only traditional *Long-Term Memory* (LTM), but any type of knowledge, including procedural and implicit perceptual knowledge. Perception, encoding, and recognition are processes that are guided by attention and are closely related to access of information in the KS^78, 79^. Access to the KS may be an event-related or a continuous process. In the latter case, the alpha band frequency range reflects continuous ‘semantic orientation’, which represents the ability to be consciously oriented in time, space, and with respect to the meaning of all entities surrounding the individual^80^. This ability requires a certain kind of attention and it is knowledge-based, which means that semantic orientation provides individuals with the ability to selectively access stored information that represents the meaning of sensory information and ‘higher order information’, such as language, mathematics, and geography^81–83^. Selective access to the KS is thought to depend largely on inhibiting task-irrelevant memory entries. Thus, periods of prolonged access should be associated with ERS, reflecting increased (i.e. synchronization) alpha band activity^84–86^.

As a result, we hypothesized to discriminate the SRK behaviours by evaluating the degree of information processing, working memory (frontal theta EEG rhythm)^16, 38, 45, 46^, procedural memory (parietal theta EEG rhythm)^43, 53^, and attention (frontal alpha EEG rhythm)^51, 64, 69, 75^ along the execution of the SRK events. The brain activations supposed to characterize the SRK levels of cognitive control have been summarized in Table 1.

**Table 1:** Hypothesized SRK characterization by means of the selected EEG rhythms. The SRK behaviours were described in terms of variation from a reference condition: *synch* – synchronization; *desynch* – desynchronization.

COGNITIVE CONTROL BEHAVIOUR	EEG RHYTHM		
	Frontal Theta	Parietal Theta	Frontal Alpha
**Skill**	*Lowest Synch*	*Lowest Synch*	*De-synch*
**Rule**	*Synch*	*Synch*	*Synch*
**Knowledge**	*Highest Synch*	*Highest Synch*	*Synch*

### Experimental Subjects

Informed consent for both study participation and publication of identifying information and images was obtained from a group of 15 ATC Experts (40.41 ± 5.54 years old), and a group of 22 ATC Students (23 ± 1.95 years old) in the final year of the course from the *École Nationale de l’Aviation Civile* (ENAC) of Toulouse (France) after the explanation of the study. In total, thirty-seven ATCOs (*Air Traffic Controllers*) were involved in the experiment. The ATCOs have been selected in order to have homogeneous experimental groups in terms of age and expertise. The experiment was conducted following the principles outlined in the Declaration of Helsinki of 1975, as revised in 2000. The study protocol received the favourable opinion from and has been approved by the Ethical Committee of the Sapienza University of Rome. The study involved only healthy participants, recruited on a voluntary basis. The ATCOs were free to accept or not to take part to the experimental protocol, and all of them accepted to participate to the study. Only aggregate information has been released while no individual information were or will be diffused in any form.

### Air Traffic Management Simulation

**Figure 2:**
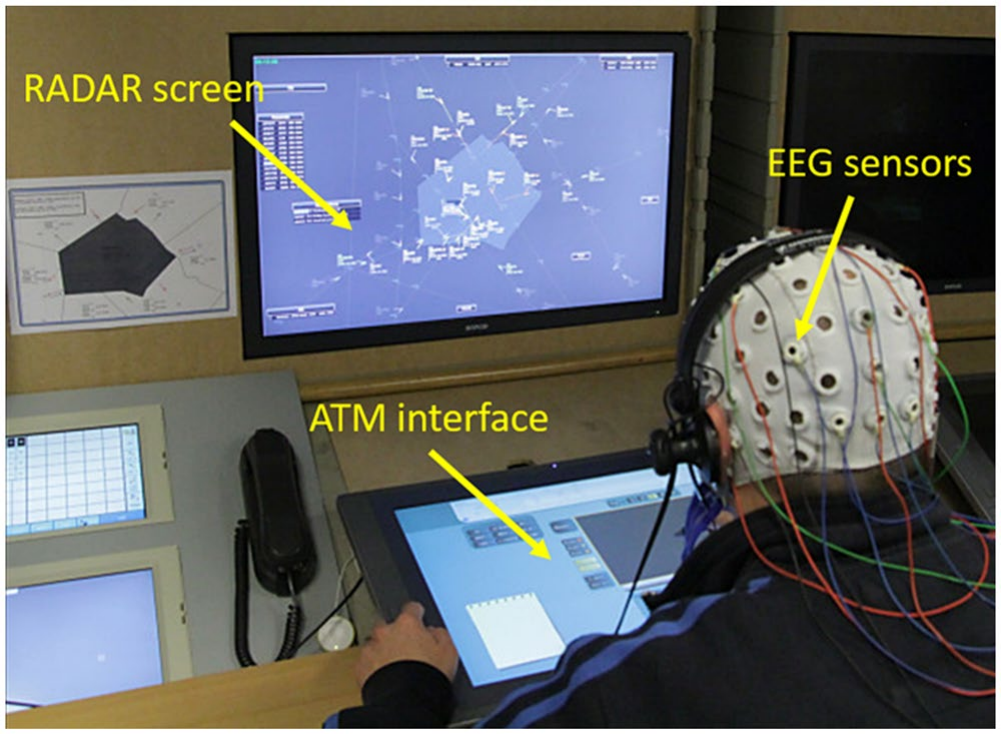
Experimental setup: ATCO working positions developed and hosted at ENAC (Toulouse, France). The ATCO’s brain activity has been recorded continuously during the execution of the ATM scenario, and the SRK events have been marked in order to recognize them within the EEG recording.

The experimental setup has been designed by HF and ATCO Experts in order to provide a high level of realism in terms of similarity to the activities and context experienced by ATCOs in their daily operational environment. The ATCOs have been asked to perform an *Air Traffic Management* (ATM) scenario using a research simulator hosted at ENAC (Fig. 2). Also, at the end of the simulation, the controllers who participated to the experiment rated the realism of the ATM scenario as “Very high”. Two Pseudo-Pilots have also been involved in the experiments to simulate real pilots, flying the aircrafts under ATCOs control, and communicating with them through proper radio sector frequencies. Although there were only two pseudo-pilots, cabin noise and slight deformation of the pilots’ voice were chosen and associated to each aircraft by a voice recognition module developed by ENAC^87^. As a result, the ATCOs heard different voices and different cabin atmospheres for each assumed aircraft. The ATM simulation scenario has been designed from real traffic samples normally used in the training program at ENAC, and it has been modified according to the objectives and needs of the experiment. The adjustments mostly impacted the routes and air-traffic within the air-space sector (adding or changing them), while the borders of the sectors were not changed. The air-traffic adjustments consisted in flights rescheduling and *flight levels* (FLs) changing, according to the *Number of aircraft*, *Traffic geometry*, and *Number of conflicts*, with the aim to define different air-traffic complexity levels, *Easy* (E), *Medium* (M) and *Hard* (H) on average (but with a changing shape over time), and simulate realistic transitions between such ATC conditions. The total duration of the scenario was 45 minutes, while the E, M and H levels lasted 15 minutes each.

### SRK Events

**Figure 3:**
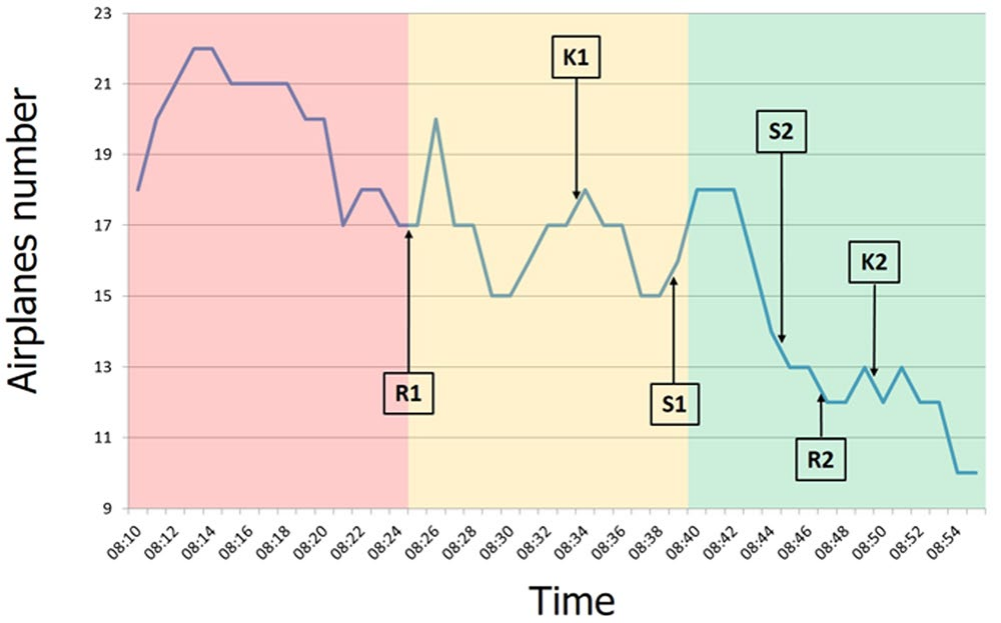
Distribution of the SRK events along the considered ATM scenario. The triplets of SRK events have been inserted in a randomized sequence in order to avoid any habituation and expectation effects. No SRK events have been inserted in the Hard condition in order to do not generate unrealistic ATC conditions.

The ATM simulation scenario included specific SRK events designed to generate the corresponding *Skill, Rule and Knowledge-based* behaviours. A *Subject Matter Expert* (SME) from the *Ente Nazionale di Assistenza al Volo* (ENAV, Rome, Italy) has been involved in order to create realistic and not disruptive SRK events during the simulation (to be limited in time and not changing the realism of the scenario). Two triplets of SRK events (S1, R1, K1, and S2, R2, K2, 6 events in total) have been inserted into the ATM scenario in a randomized sequence, as reported in Fig. 3, in order to avoid any habituation and expectation effects.

In particular, the SRK events have been inserted into task phases with different difficulty levels (3 SRK events in the E condition, and 3 events in the M condition) with the aim to check the capability of the selected brain features of characterizing the three cognitive control behaviours in realistic ATM settings. In fact, since the ATM scenario has been designed with the aim to achieve high air-traffic realism and conditions, the task difficulty and complexity kept varying along the time and, as a consequence, the different SRK events happened within different difficulty levels. Therefore, if the chosen brain features were, for example, related only to the task difficulty or workload, it could be not possible to discriminate the SRK events. The events have not been inserted into the H part of the ATM scenarios, since the introduction of external events could increase the complexity to an unacceptable level, generating too much disruption on the controlling activity and possibly invalidating part of the recording and of the realism of the task. The design of the SRK events has been the following. The *Skill* (S) events were basically interactions with the interface, during the task execution. Controllers were asked, by showing them a sheet with the instructions, to visualize the distance between two aircrafts (Distance event) or to display the *Flight Plan Level* (FPL) trajectory of each aircraft present in the controlled sector (Display FPs). The *Rule* (R) events were mainly control-tasks and conflicts-resolutions, during which Controllers were also performing skill-events (interaction with the interface). In the “Conflict event”, Controllers had to detect and solve a conflict by using the menu of the interface, assign new altitudes and headings, and eventually to call the pilots asking information about their FPLs. The hypothesis was that conflict detection task represented a familiar situation for ATCOs. Therefore, Controllers should recognize the correct procedures and usual solutions and then apply them to solve the conflict. The *Knowledge* (K) events integrated in the ATM scenario represented more unfamiliar and unusual situations. They generated a certain level of uncertainty, leading the Controllers to require time to analyse the situation and to find out the proper procedure to cope with the unexpected event. In other words, the ATCOs initially had to analyse the situation, identify the unusual situations, and then come back to the rule-based level for adopting the right procedures. In the first knowledge-based event, an aircraft deviated from the route filled in the initial FPL. An alarm was displayed on the Radar interface with the aim to make the ATCO focusing on the aircraft (a/c), checking its manoeuvres, and detecting that something was happening. Once contacted, the pilot claimed to be on the right FPL. Controllers needed to understand if there was a problem with the system or a pilot’s error. In the second knowledge-based event (*Unidentified Flying Object* - UFO), the Pseudo-Pilot reported an unknown-traffic detected by the *Traffic Collision Avoidance System* (TCAS) and a TCAS resolution advisory to avoid a mid-air collision. This unknown aircraft has not been displayed on the Controller’s radar image, who had been supposed to understand the situation and to ask additional information to the pilot of the considered aircraft. After the avoidance manoeuvre (e.g. descent), the pseudo-pilot had to ask for his previous flight-level, which never changed on Controller’s HMI because the pseudo-pilots were instructed to act during this event. The Controller could not observe neither the aircraft responsible of the TCAS advisory nor the implementation of the avoidance manoeuvre. In Table 2, we reported detailed descriptions of the six SRK events, in the proposed order, to show how couples of same type of events were not exactly the same, in terms of requested actions to perform, in order to make the results more robust to possible confounds on brain activations while accomplishing the SRK events. Finally, along the execution of the SRK events, SMEs (sat behind the ATCOs) checked if the Controllers performed correctly the considered events. The SMEs confirmed that all the involved Controllers, both Experts and Students, did execute correctly the proposed SRK events.

**Table 2:** Detailed descriptions of the six SRK events designed to induce Skill, Rule and Knowledge behaviours in the Controllers during the execution of the ATM scenario.

Type of Event	Description	Implementation	Expected Controller Actions
**R1** (*Conflict*)	Controllers have to detect and solve a conflict, using the *pie menu* to assign new altitudes and headings.	Flight DLH27R calls: “DLH27R, I would like to climb to FL370 to avoid a heavy turbulence”.	Controller would observe his radar screen and then he would open the *pie menu*. Clicking on *Confirm Flight Level* (CFL), he would assign the new required altitude. Then, to avoid a conflict between flight DLH27R and flight AFR629, he would use the *pie menu* to change the flight AFR629 altitude to FL360, and eventually, he would call the pilots to ask information about their FPLs.
**K1** (*Deviation*)	Controllers are asked to face with unexpected situation, in which an aircraft changes its route respect to the declared FPL. Controllers have to detect the manoeuvres and check the *aircraft* (a/c) FPL on the RADAR interface to evaluate if something is going wrong.	Flight FGJNH would turn to HARRY instead of flying the original *flight route*.	After the detection of the aircraft unexpected manoeuvre, Controller would call flight FGJNH asking for explanations. Then he would check the original FPL to evaluate if this latter is correct or if something is going wrong. After flight FGJNH call, once clarified the situation, he would give the pilots instructions to move back to the original FPL.
**S1** (*Distance*)	Controllers were asked to measure the distance between two aircrafts using the “*alidade”* tool. The *alidade* tool is used to measure the distance between two points on the radar image. The user selects the origin of the alidade and the tool will draw a line which follows the cursor and indicates the distance from the origin.	SME asks the Controller to measure the distance between flight NJE123 and flight IBE692.	Controller would use the *alidade* tool, clicking on flight NJE123 and dragging the cursor to Flight IBE692; the system would provide the distance between such aircrafts.
**S2** (*Display FPs*)	SME will trigger the event showing the Controller a sheet with the instruction to visualize the *Flight Plan* (FPL) trajectory of each assumed aircraft. Controller have then to use the *pie menu* of the WACOM interface and select the *route option* for each aircraft.	SME shows the Controller a paper with the following instruction: “Please visualize the FPL trajectory for each assumed aircraft on your radar screen. This is just a system check. Thank you”.	Controller would open the *pie menu* and select the *route option* for each aircraft.
**R2** (*Conflict*)	Controller has to detect and solve a conflict, using the *pie menu* to assign new altitudes and headings.	Flight ICE873 calls: “ICE873, I would like to descend to FL370 to avoid a heavy turbulence”.	Controller would observe his radar screen and then he would open the *pie menu*. Clicking on *CFL* option, he would assign the new required altitude, and eventually, he would call the pilots to ask information about their FPLs. Expected conflict with flight TAP369 at FL380.
**K2** (*UFO*)	Controller is asked to verify an unknown traffic detected by the *Traffic Collision Avoidance System* (TCAS), to avoid a mid-air collision. After the avoidance manoeuvre, pilots report a different FPL with respect to what provided by the RADAR interface.	Flight RAE1789 calls: “I have a traffic advisory. I’ll call you back” After 10 seconds, with emphasis: “TCAS resolution, we are descending”. After 10 seconds, a/c will call “cleared of conflict, we are at FL380”. (without really change the FPL).	Controller would verify this *unknown* traffic on his RADAR interface, but he would not see any aircraft. Then Controller would eventually check the RADAR interface to verify if there is some activated filters. After a/c descent, the Controller would still see FL390 on the a/c label, probably would ask to a/c to check and confirm its FPL.

### Physiological Signals Recording and Pre-Processing

The neurophysiological signals have been recorded using the digital monitoring *BEmicro* system (EBNeuro system, Italy). The nine EEG channels (*FPz, F3, Fz, F4, AF3, AF4, P3, Pz* and *P4*) have been collected with a sampling frequency of 256 (Hz). All the EEG electrodes have been referenced to both the earlobes, grounded to the mastoids, and the impedances of the electrodes were kept below 10 (kΩ). The acquired EEG signals have been digitally band-pass filtered by a 5^th^ order Butterworth filter (low-pass filter cut-off frequency: 30 (Hz), high-pass filter cut-off frequency: 1 (Hz). The eye-blink artifacts have been removed from the EEG using the *Reblinca* method^88^. In particular, with respect to other regressive algorithms (e.g. Gratton method^89^) the *Reblinca* method presents the advantages to preserve EEG information in blink-free signal segments by using a specific threshold criterion that automatically recognize the occurrence of an eye-blink, and only in this case the method corrects the EEG signals. If there is not any blink, the method does not introduce any changes into the EEG signal. In addition, the *Reblinca* method does not require any EOG signal(s). The EEG signal has been then segmented in 2 second-epochs, shifted of 0.125 (sec), with the aim to have both a high number of observations in comparison with the number of variables, and to respect the condition of stationarity of the EEG signal^90^. In fact, the latter one is necessary in order to proceed with the spectral analysis of the signal. For other sources of artifacts, specific procedures of the EEGLAB toolbox have been applied^91^. In particular, three methods have been used: the *threshold criterion*, the *trend estimation* and the *sample-to-sample difference*. In the threshold criterion an EEG epoch has been marked as “artifact” if the EEG amplitude was higher than ±100 (μV). In the trend estimation, the EEG epoch has been interpolated in order to check the slope of the trend within the considered epoch. If such slope was higher than 3 (µV/epoch) (non-physiological variation), the considered epoch has been marked as “artifact”. The last check calculated the difference between consecutive EEG samples. If such difference, in terms of amplitude, was higher than 25 (μV), it meant that an abrupt variation (non-physiological) happened, thus it was marked as “artifact”. At the end, the EEG epoch marked as “artifact” have been removed with the aim to have a clean EEG dataset from which estimating the brain parameters for the different analyses. The *Power Spectral Density* (PSD) has then been estimated by using the *Fast Fourier Transform* (FFT) in the EEG frequency bands defined for each subject by the estimation of the Individual *Alpha Frequency* (IAF) value^92^. By segmenting the EEG signal, we achieved an average number of 224 epochs, for a 30 second-long event, with an average rejection rate of 17.3 ± 13.6%. In addition, by using 2 second-long *Hanning* windows for the PSD estimation, we obtained a frequency resolution of 0.5 (Hz). Thus, according to the definition of theta [IAF-6 ÷ IAF-2] and alpha [IAF-2 ÷ IAF + 2] EEG bands, we obtained 17 frequency bins, i.e. 17 PSD values for each EEG channel. Furthermore, the *Baseline* (brain activity during a minute of rest conditions, that is, closed eyes - OC) and the *Reference* (ATCOs looked at the radar screen without reacting for 3 minutes, where two no-colliding airplanes have been presented - REF) conditions have been recorded before starting with the ATM simulations. The OC condition has been used for the IAF estimation, while the REF condition has been used to evaluate the PSDs variations, with respect to the considered experimental condition, within the S, R and K events in terms of z-score^93^ values. The results proposed in the following sections will be represented as *z-score* values, thus values (y-axis) of zero mean that there are no differences, in terms of PSD, between the considered experimental condition (S, R and K) with respect to the REF one. Positive z-score values mean synchronization of the considered EEG rhythm (PSD increment), while negative z-score values mean desynchronization of the considered EEG rhythm (PSD decrement).

### SRK Neurophysiological Characterization

The *Power Spectral Density* (PSD) has been estimated for the different brain features (frontal and parietal theta, and frontal alpha EEG rhythms), and the analysis of their spectral information has been performed in order to assess if they could be used to achieve the proposed goal. One-way repeated measures ANOVAs have been performed for each brain rhythm with the SRK, (3 levels: Skill, Rule and Knowledge) as *within* factor, and with the PSD as independent variable.

### SRK Discrimination: Machine-Learning Analysis

The classification algorithm *automatic stop StepWise Linear Discirminant Analysis* (asSWLDA)^17, 49^ has been used to select the most relevant brain spectral features. In particular, the frontal and parietal theta, and frontal alpha EEG rhythms have been used for the calibration of the asSWLDA^94^ to characterize and discriminate the different cognitive control levels (SRK) during the execution of the ATM simulation. Therefore, the asSWLDA algorithm has been calibrated by using brain features (i.e. PSD epochs) extracted within the considered spectral domain (frontal and parietal theta, and frontal alpha) from one triplet of SRK events (S1, R1, K1), and then tested on the remaining triplet (S2, R2, K2), and vice-versa. For each testing triplet, the *Area Under Curve* (AUC) values have been calculated of the *Receiver Operating Characteristic* (ROC)^95^ by considering couples between SRK distributions. The AUC values related to the discrimination accuracy between the three couples of conditions (S vs R, S vs K, R vs K) have been calculated and analysed for each ATCO. In order to test the effectiveness of the algorithm, and that the SRK discrimination would not be due to chance, for each couple of conditions (S vs R, R vs K, S vs K) we have compared the AUC distributions obtained from the experimental data of the ATCOs (*Measured AUC*), with a random distribution calculated for each specific couple (*Random AUC*), situation corresponding to the random classification. In particular, we performed all the possible permutations along both the calibrating and testing dataset^96, 97^. In this regard we shuffled the SRK labels for the ATCO Experts related data, and then we calculated the AUC values between couples of conditions (e.g. S vs R) considering two cases: one in which the right labels have been used (*Measured AUC*), and the other in which the labels have been randomly set (*Random AUC*). The *Random AUC* distributions have been compared with the *Measured AUC* by using three two tailed student t-tests (α = 0.05), in order to demonstrate the reliability of the algorithm. For the ATCO Students, we were unable to perform a three-class analysis, because most of them missed the first *knowledge*-based event (*Deviation*). In this regard, we have considered only the *rule* and *skill* conditions. In particular, the asSWLDA has been calibrated by using one couple (S1, R1) and the discrimination accuracy (AUC values) has been tested on the remaining couple (S2, R2), and *vice-versa*. As consequence, the AUC distributions (*Measured AUC*, and *Random AUC*) have been calculated only for the “S vs R” comparison.

### Comparison between ATCO Experts and Students

Two-tailed unpaired t-tests (α = 0.05) have been performed to compare the discrimination accuracy (i.e. AUC) between the skill and the rule conditions (the knowledge event was missed as quoted above) between the two groups (ATCO Experts and ATCO Students). The hypothesis was that the ATCO Students were not as skilled as the ATCO Experts, so that the skill-based events (that should be characterised by completely automated activities) could require a brain activation and attention as for the rule-based events. In this regard, the asSWLDA has been calibrated by using one couple of conditions (S1, R1), and the AUC values have been calculated on the remaining couple (S2, R2) and vice-versa, both for the ATCO Experts and Students. In addition, one-way ANOVAs have been performed on the frontal and parietal theta, and frontal alpha PSDs with the aim to assess eventual differences between the two groups (*between* factor RANK; 2 levels: Experts and Students). Before every statistical analysis, the *z-score* transformation^98^ has been used to normalize the data. Furthermore, the most common brain features selected by the asSWLDA, within the single EEG band and in total, have been analysed and compared between the two groups. In particular, we assigned “1” to the selected features, and then we summed up the values over the cross-validation and ATCOs, separately for the Experts and Students, to finally calculate the average rate by which each feature was picked for the SRK events discrimination. For example, if a frequency bin, within the frequency range “*theta* + *alpha band*”, was selected 5 times for all the ATC Experts, it was then divided by the total number of cross-validations (2 cross-validations) multiplied by the number of the considered ATCOs (15 Experts). Therefore, the final percentage corresponding to such a bin was (5/(2 * 15)) * 100 = 16.67%. Two-tailed unpaired t-tests have then been performed to assess possible differences between the ATCO groups in terms of selected brain features.

## Results

### SRK Neurophysiological Characterization

**Figure 4:**
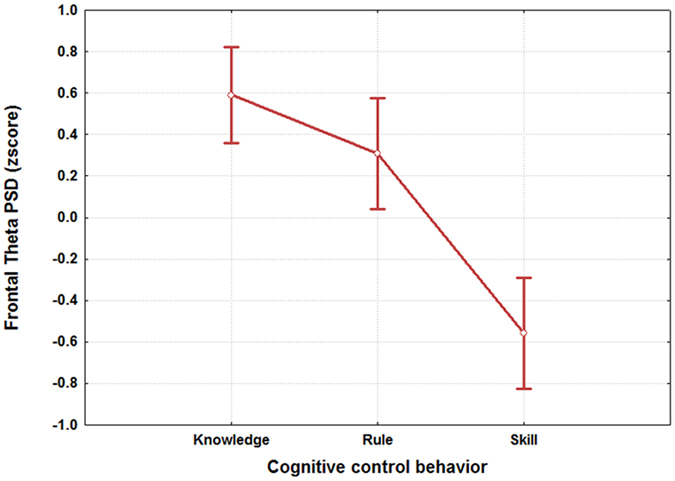
The figure reports the results of the ANOVA analysis on the frontal theta PSD with the factor “SRK” of 3 levels (Skill, Rule and Knowledge). The results showed that such brain feature could be used as SRK discriminant brain feature, as its PSD values were significantly different (p = 0.000001) between the S, R and K levels.

The results of the ANOVA on the frontal theta (Fig. 4) PSDs showed that this brain features changed significantly (F(2, 64) = 17.01; p < 10^−5^) among the S, R and K events, thus it could be used for the SRK discrimination. In particular, the post-hoc test reported high discriminability of the skill event with respect the other two (p < 0.00006), and the difference between the knowledge and the skill events (p < 0.0002). On the contrary, the rule and the knowledge did not differ significantly (p = 0.3).

**Figure 5:**
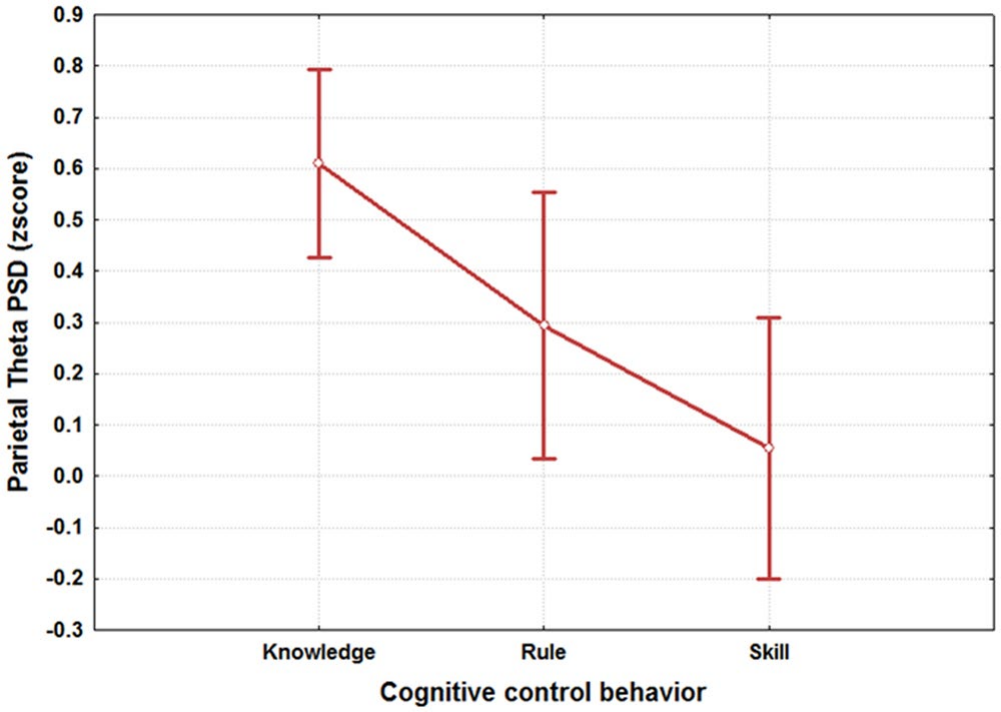
The figure reports the results of the ANOVA analysis (CI = 0.95) on the parietal theta PSD with the factor “SRK” of 3 levels (Skill, Rule and Knowledge) The results showed that such brain feature could be used as SRK discriminant brain feature, as its PSD values were significantly different (p = 0.02092) between the S, R and K levels.

The ANOVA performed on the parietal theta PSD (Fig. 5), demonstrated that it significantly changed across the SRK levels (F(2, 64) = 4.11; p = 0.021). The post-hoc showed that the parietal theta PSD could be used to discriminate accurately the skill from the knowledge level (p < 0.002). On the contrary, the parietal theta PSD did not change significantly between the rule – knowledge, and skill – rule levels (p > 0.25).

**Figure 6:**
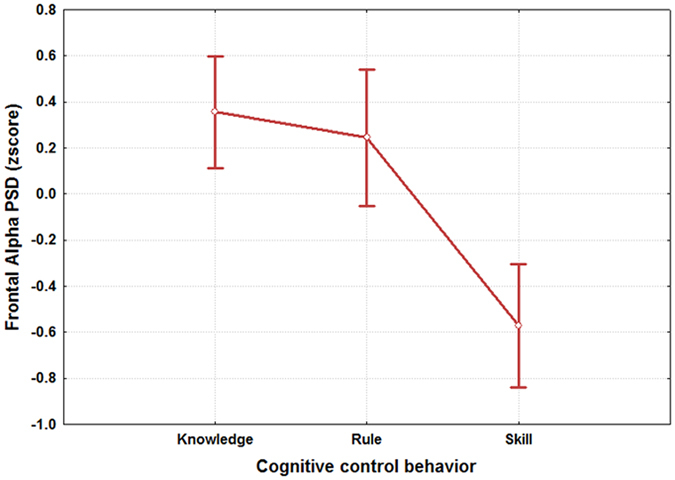
The figure reports the results of the ANOVA analysis (CI = 0.95) on the frontal alpha PSD with the factor “SRK” of 3 levels (Skill, Rule and Knowledge). The results showed that such brain feature could be used as SRK discriminant brain feature, as its PSD values were significantly different (p = 0.00006) between the S, R and K levels.

The ANOVA on the frontal alpha PSD (Fig. 6) reported a significant effect in the SRK discrimination (F(2, 64) = 11.48; p < 10^−4^), and the post-hoc test showed that the frontal alpha rhythm could be used to differentiate either the skill from the rule (p = 0.0003) and knowledge levels (p = 0.0002). On the contrary, the frontal alpha PSD did not change significantly between the knowledge and rule levels (p > 0.8).

### SRK Discrimination: Machine-Learning

**Figure 7:**
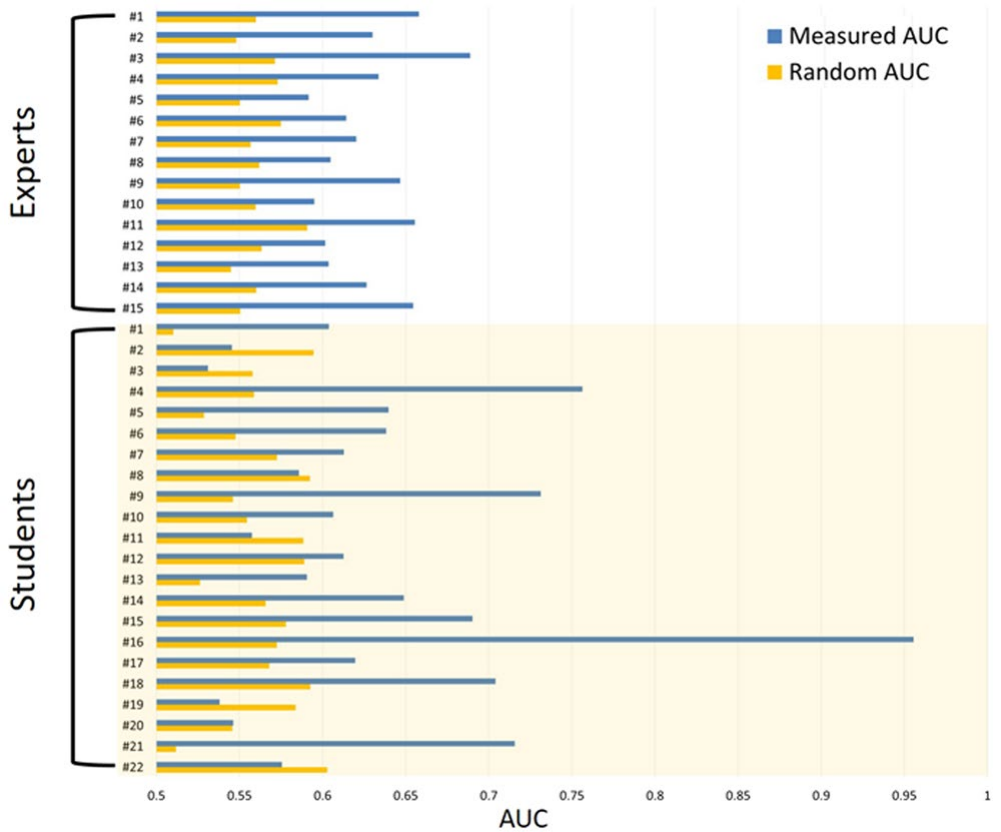
Bars plot of the averaged Measured AUC (blue bars) across all the possible labels combinations with respect to the Random AUC (orange bars) for each ATCO. In particular, the AUC values of the Experts are reported on the top part of the image, while those of the Students are reported on the bottom.

As mentioned above, the asSWLDA was calibrated by using one triplet (S1, R1, K1) and the discrimination accuracy (AUC) values were calculated by testing it on the remaining triplet (S2, R2, K2), and vice-versa. In particular, for each couple of conditions (S vs R, R vs K, S vs K) we shuffled the SRK labels and then we calculated the AUC values considering two cases: one in which the right labels have been used (*Measured AUC*), and the other in which the labels have been randomly set (*Random AUC*). Referring on the results reported in the previous sections, the frontal and parietal theta, and frontal alpha EEG rhythms were defined as the frequency domain in which the asSWLDA classification model had to select the most significant features to discriminate the three cognitive control behaviours (SRK) from the chance level. Figure 7 reports the *Measured AUC (blue bars)* from the *Random AUC* (orange bars) for each ATCO (Experts on the top part of the image) averaged across all the possible labels combinations.

### ATCO Experts

**Figure 8:**
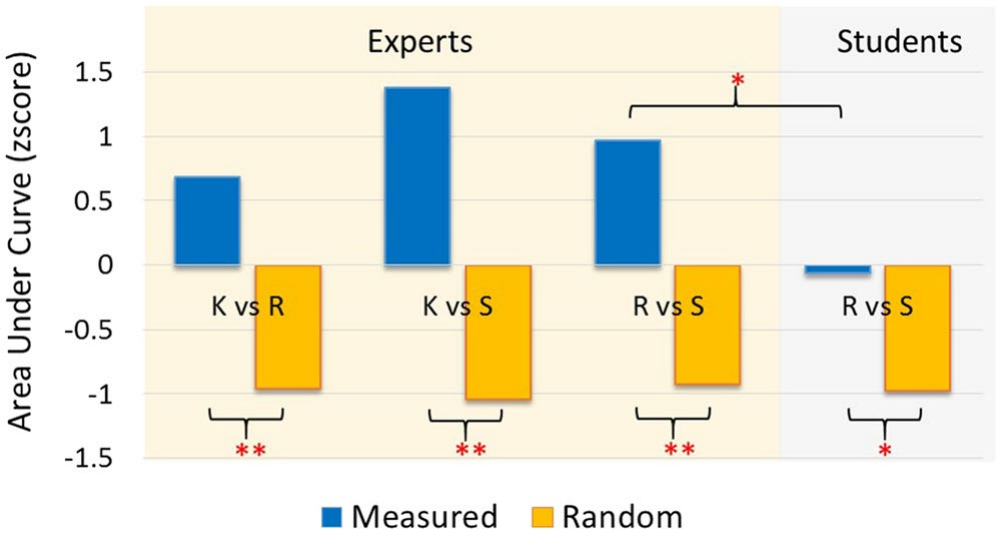
Bars plot related to the zscore-normalized Measured AUC (blue bars) distributions and the Random AUC (red bars) distributions, achieved by the ATCO Experts (left side) and Students (right side), referred to the discrimination accuracy between the three couples of conditions (S vs R, S vs K, R vs K) for Experts, and S vs R for Students. The three cognitive control behaviours could be significantly discriminate for both the groups (p < 0.05). In addition, the results show how the S and R behaviours were significantly (p < 0.05) more discriminable for the Experts than for the Students (p < 0.05).

The two-tailed paired t-tests (α = 0.05) showed that all the *Measured AUC* (blue bars) distributions were significantly higher (left side of Fig. 8) with respect to the *Random AUC* (orange bars) distributions (all p < 0.002).

### ATCO Students

The two-tailed paired t-test (α = 0.05) showed that also for the ATCO Students all the *Measured AUC* (blue bars) distributions were significantly higher (right side of Fig. 8) than the *Random AUC* (orange bars) distributions (all p < 0.05).

### Comparison between ATCO Experts and ATCO Students

Figure 8 shows also the differences between the two ATCO groups in terms of zscore-normalized AUCs. In particular, the ATCO Experts exhibited a significant higher (p < 0.05) discrimination accuracy with respect to the ATCO Students. The trend may indicate how the cognitive control behaviours were better discriminable for Experts than for Students.

**Figure 9:**
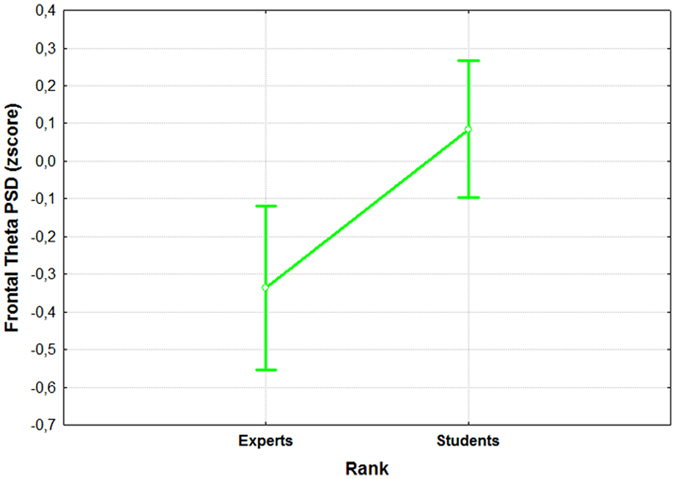
The figure reports the results of the ANOVA analysis (CI = 0.95) on the frontal theta PSD with the factor “RANK” of 2 levels (Experts and Students). The results showed that the two groups were statistically different (p = 0.005) in terms of activation of the frontal theta rhythm when facing the same SRK events.

**Figure 10:**
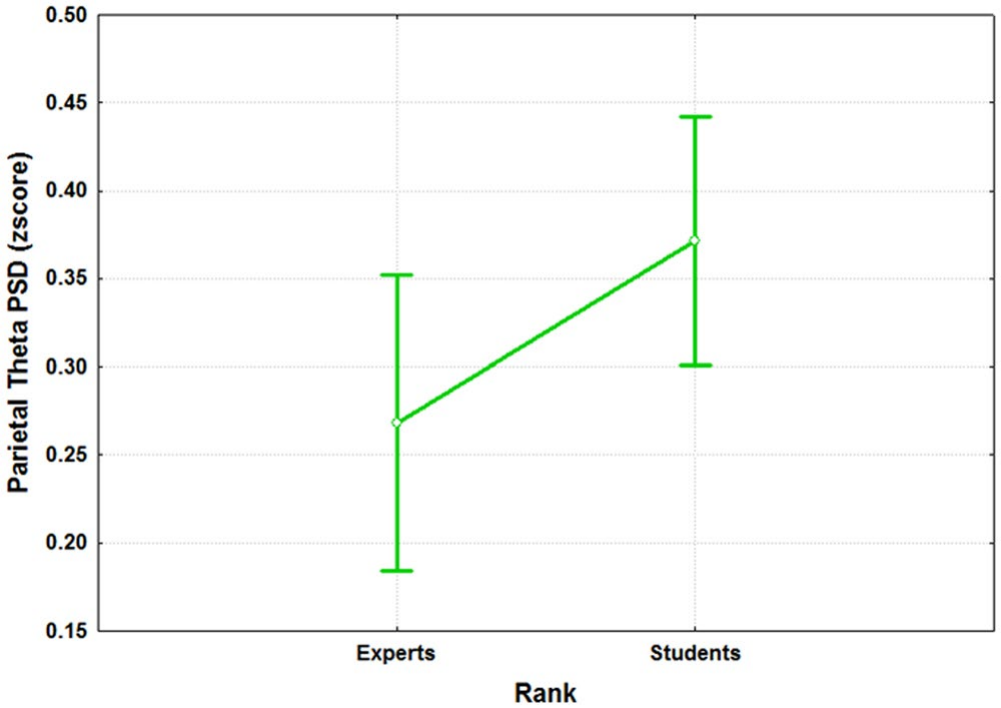
The figure reports the results of the ANOVA analysis (CI = 0.95) on the parietal theta PSD with the factor “RANK” of 2 levels (Experts and Students). The results showed that the two groups were statistically different (p = 0.0023) in terms of activation of the parietal theta rhythm when facing the same S, R and K events.

**Figure 11:**
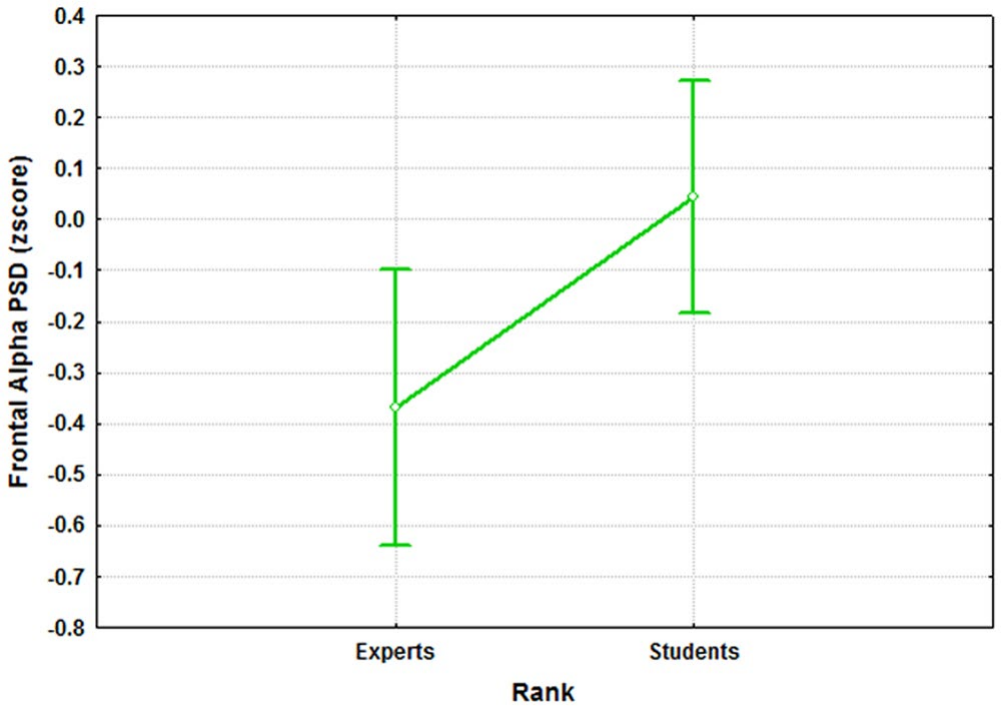
The figure reports the results of the ANOVA analysis (CI = 0.95) on the frontal alpha PSD with the factor “RANK” of 2 levels (Experts and Students). The results showed that the two groups were statistically different (p = 0.024) in terms of activation of the frontal alpha rhythm when facing the same S, R and K events.

In addition, the results performed on PSD values showed that the considered brain features could be used to distinguish the level of expertise (Expert vs Student) with significant reliability (all p < 0.03). In fact, the one-way ANOVAs showed significant differences between the group of ATCO Experts and ATCO Students in terms of brain activations (Figs 9, 10 and 11).

In particular, the ATCO Students showed both higher frontal and parietal theta synchronization, respectively (F(1, 32) = 9.14; p = 0.005) and (F(1, 32) = 10.97; p = 0.0023), and a lower (F(1, 32) = 5.62; p = 0.024) frontal alpha desynchronization than the ATCO Experts.

### Selected Brain Features

**Figure 12:**
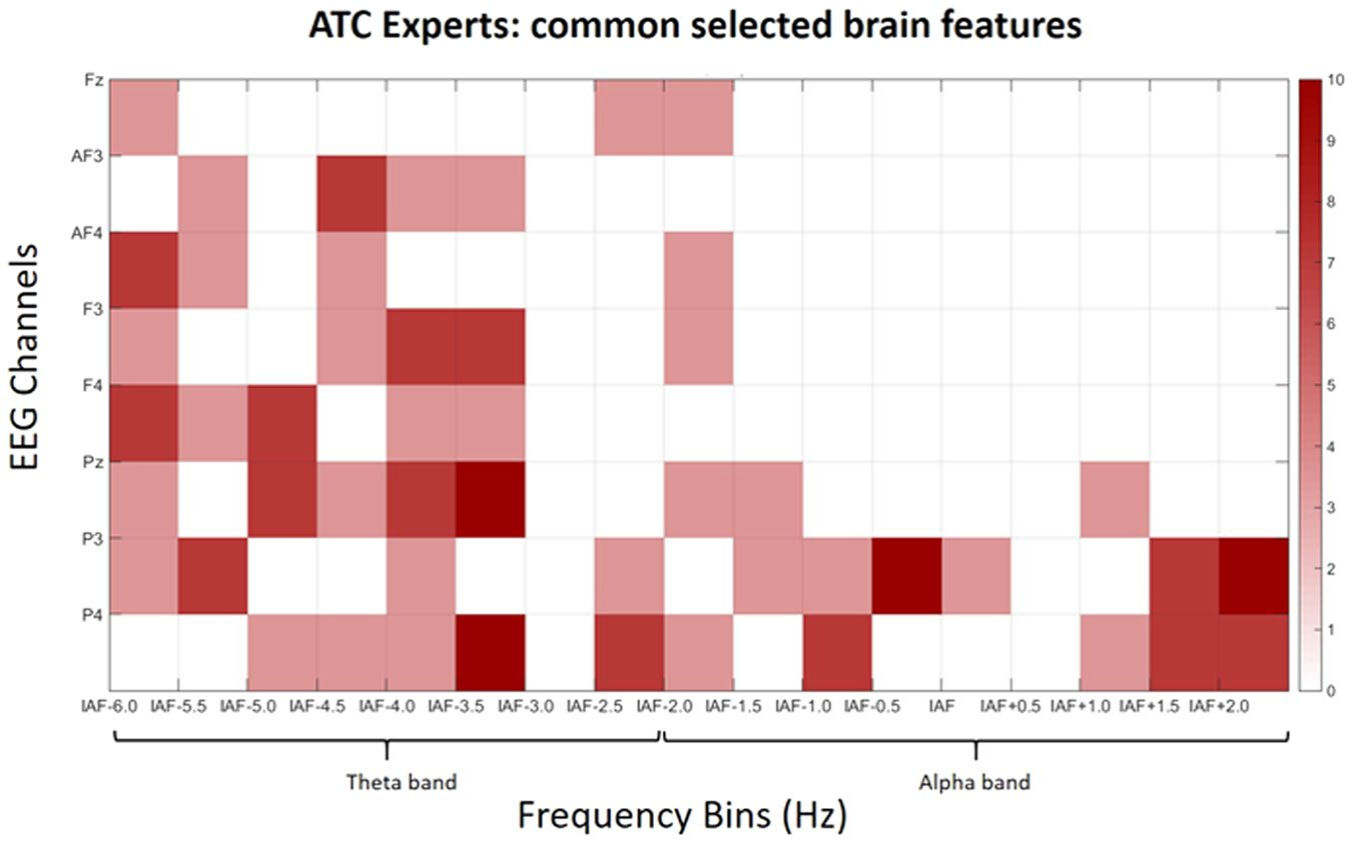
Percentages related to the brain features most commonly selected by the asSWLDA across the ATCO Experts for the SRK-based cognitive control behaviours discrimination. White color means that brain features have not been selected at all. On the contrary, the red colors means that the brain features were selected more frequently.

**Figure 13:**
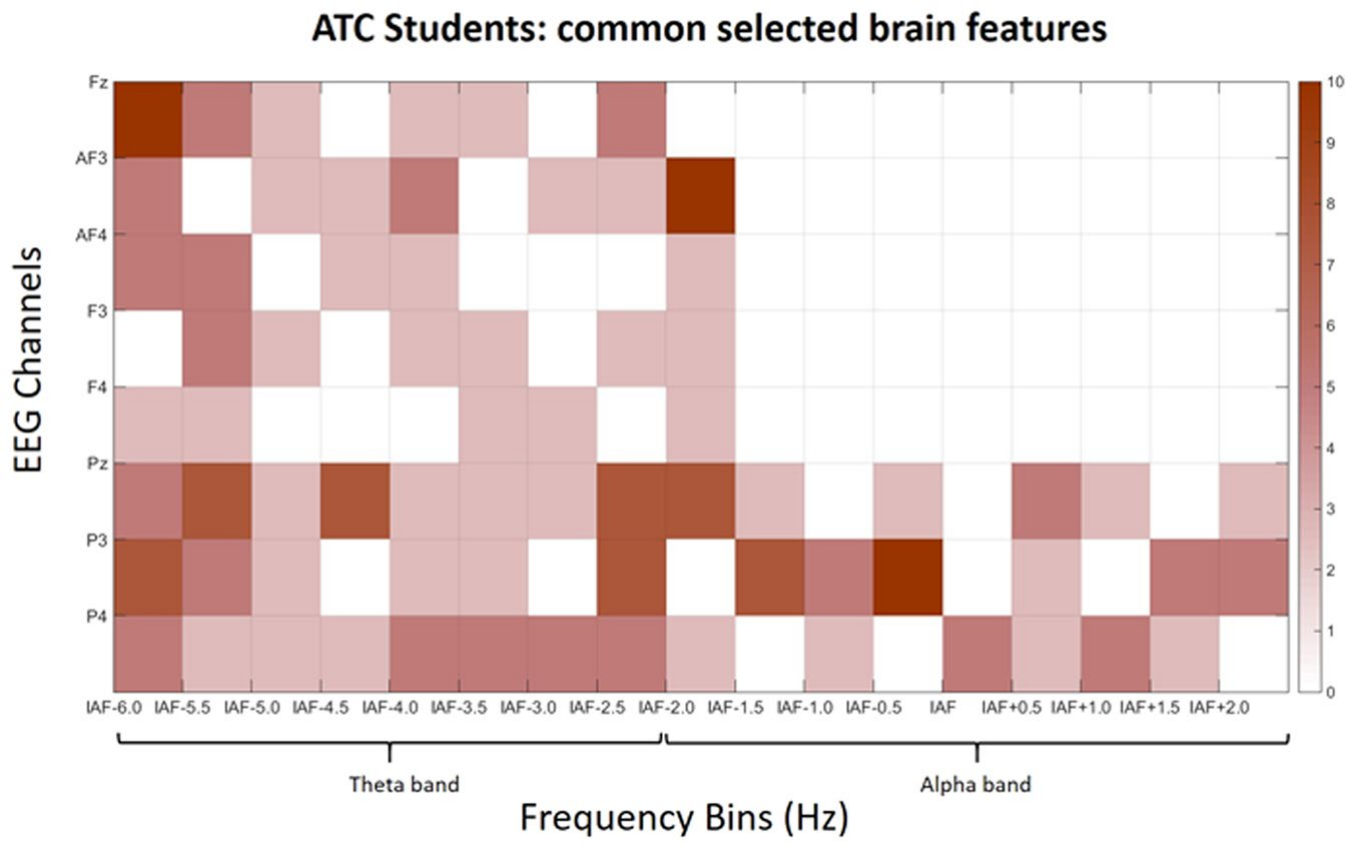
Percentages related to the brain features most commonly selected by the asSWLDA across the ATCO Students for the SRK-based cognitive control behaviours discrimination. White color means that brain features have not been selected at all. On the contrary, the red colors means that the brain features were selected more frequently.

In Figs 12 and 13, the selected brain features for the ATCO Experts and ATCO Students are respectively reported. The results showed that, on average, the selected brain features were 5.15 for the ATCO Experts, and 5.6 for the ATCO Students with no general statistical difference between them (p = 0.59). However, by the inspection of the figures, it can be highlighted that the selected features over the frontal areas of the Experts were lower than for the Students. Thereafter, two two-tailed unpaired t-tests have been performed separately on the features selected within the frontal EEG channels, and within the parietal EEG channels. The results showed a clear trend (even if not strictly significant p = 0.07) in the number of selected features over the frontal areas, since they were significantly higher for the ATCO Students (Fig. 13) than for the ATCO Experts (Fig. 12), while no difference were found among the selected parietal features (p = 0.63).

## Discussion

Several studies^28, 33, 99–101^ tried to model the different information processing procedures based on the *Skill*, *Rule* and *Knowledge* cognitive control behaviours from a HF perspective. Despite such studies, there are not evidences in which these cognitive control levels have been taken into account from a neurophysiological point of view, for example, by considering the EEG variations between the different SRK levels. The multi-disciplinary approach^102^ applied in this work started with an extensive literature review that identified the main cognitive processes related to the S, R and K-based behaviours. They were characterized considering only cognitive processes linked to the level of automatism when dealing with the different events. In other words, we sought features linked to information processing, decision making, working memory, and attention, without considering motor brain features, to avoid SRK behaviours discrimination depending on different amount of movements (hands and/or feet) during the execution of the ATC activities. Furthermore, the ATM scenario was designed as realistic as possible, and validated by ATCO and HF experts. Additionally, all the SRK events were inserted within different difficulty levels continuously changing over time to investigate whether the selected brain features were appropriate to discriminate the cognitive control behaviours despite the task difficulty or workload variations. The considered brain features for the SRK discrimination were the frontal and parietal theta, and the frontal alpha EEG rhythms. In fact, the frontal theta rhythm is linked to decision–making processes and working memory, therefore it is directly correlated to the amount of information and stimuli to process. Hence, the more complex the task is, the higher the theta synchronization over the frontal brain areas is. The second one, the parietal theta, is correlated to the procedural memory, and the more sustained the memory consolidation is, the higher its synchronization over the parietal brain areas is. The third EEG rhythm, frontal alpha, is correlated to the attentional level, and it has been demonstrated that the higher the attention is, the more the desynchronization of the alpha activity over the frontal brain areas is. The analysis of the frontal and parietal theta, and frontal alpha PSDs across the SRK levels confirmed that these brain features changed significantly across the SRK levels (Figs 4, 5 and 6). In fact, as expected, the frontal theta PSD increased with the complexity of the task, in particular especially in correspondence of the *knowledge* than in the *skill* events. The parietal theta PSD, representative of memory consolidation, increased less during automatic activities (i.e. skill events) than during more procedural or planning activities, that is *knowledge* and *rule* events. Similarly, the frontal alpha EEG rhythm, correlated to attentional level, desynchronised more during automatic actions than during activities where access to *Knowledge System* (KS) was required, as *rule* and *knowledge* events.

We would like to underline that by the analysis of the PSDs was not possible to discriminate all the SRK levels, but only the K, R behaviours from the S one, and not the K from the R ones. On the contrary, by using a machine-learning approach, we were able to discriminate significantly all the three cognitive control behaviours. In addition, the considered EEG features allowed to highlight the differences between the ATCO Experts and ATCO Students groups, in terms of expertise (Figs 7, 8, 9, 10 and 11). In this regard, the ATCO Experts showed higher memory consolidation and both lower attentional request and demand on executive controls than the ATCO Students. Such brain features were then used to calibrate the asSWLDA classification algorithm, to investigate the possibility to discriminate the three cognitive control levels during the execution of the realistic ATM scenario. The results confirmed that the asSWLDA was able to discriminate significantly the SRK conditions, as all the measured AUC distributions were significantly higher (p < 0.05) than the chance level (Fig. 7). Additionally, the analysis on the amount of brain features selected by the asSWLDA for each group demonstrated how the ATCO Students showed higher frontal brain activation than the ATCO Experts in dealing with the same SRK events (Figs 12 and 13). Therefore, we may conclude that such difference supports the higher information processing and working memory activity for the Students, with respect to the Experts, attributable to the different degree of expertise.

A limitation of the study has also to be discussed. In fact, because of the high realism of the ATM scenario, it was not possible to strictly design “pure” SRK events. Therefore, in combination with the SRK events, ATC activities were also performed. In order to investigate the possibility to measure and quantify SRK events in realistic settings, the HF and ATCO experts properly inserted the SRK events within ATM conditions of different complexity, where the difficulty profile of the task changed over time to simulate realistic air-traffic situations. Therefore, we tested if we were able to discriminate the SRK behaviours when the difficulty of the faced condition changed (i.e. different workload demand). Consequently, in the next studies we shall design experimental protocols by using controlled tasks in order to better simulate and induce pure *skill*, *rule* and *knowledge-based* behaviours into the users. This design will decrease the realism of the simulation, but it could allow to confirm and refine the promising findings of this work.

## Conclusions

In this study, it has been demonstrated that it was possible to assess, with a high reliability, the ATCOs’ cognitive control behaviour (SRK) while performing realistic *Air-Traffic-Management* (ATM) scenario by means of specific brain features. Such brain features were selected with the aim to characterize the different SRK levels in terms of “cognitive automatism”, minimizing possible influences of motor activity (hands or feet movements). The results showed that i) the proposed neurometric (frontal and parietal theta, and frontal alpha EEG rhythms) could be valid metrics to investigate and objectively analyse the user’s cognitive control behaviour (SRK); ii) the asSWLDA was able to discriminate significantly the cognitive control behaviour for both the ATCOs groups (Experts and Students); and iii) the proposed methodology could be applied to assess differences in expertise between groups.

The proposed method could be potentially used as a *Human Factors tool* during the design phase of new solutions, as a *Training tool* to evaluate the level of learning and expertise gained by the user, and as a *Monitoring tool* to be used during operations (e.g. crew monitoring on a/c or ATC rooms). Additionally, it could provide objective information for a more precise prediction of the ways the operators may possibly fail in achieving their goals. This is particularly important for the *Safety* and *Human Factors* fields, whose goals are enhancing people performance, avoiding error-prone situations, and neuroergonomics designing of environments^4, 103^.
